# Effects of Dietary Fibre from the Traditional Indonesian Food, Green Cincau (*Premna oblongifolia* Merr.) on Preneoplastic Lesions and Short Chain Fatty Acid Production in an Azoxymethane Rat Model of Colon Cancer

**DOI:** 10.3390/ijms19092593

**Published:** 2018-08-31

**Authors:** Samsu U. Nurdin, Richard K. Le Leu, Arturo Aburto-Medina, Graeme P. Young, James C. R. Stangoulis, Andy S. Ball, Catherine A. Abbott

**Affiliations:** 1 College of Science and Engineering, Flinders University, GPO Box 2100, Adelaide 5001, Australia; samsu.udayana@fp.unila.ac.id (S.U.N.); james.stangoulis@flinders.edu.au (J.C.R.S.); 2 Flinders Centre for Innovation in Cancer, Flinders University, GPO Box 2100, Adelaide 5001, Australia; Richard.LeLeu@sa.gov.au (R.K.L.L.); graeme.young@flinders.edu.au (G.P.Y.); 3 School of Science, RMIT University, P.O. Box 71, Bundoora, Victoria 3083, Australia; a.arturo1309@gmail.com (A.A.-M.); andy.ball@rmit.edu.au (A.S.B.)

**Keywords:** aberrant crypt foci, antioxidant, azoxymethane, colorectal cancer, dietary fibre, green cincau, gut microbiota, short chain fatty acids

## Abstract

Green cincau (*Premna oblongifolia* Merr.) is a traditional food of Indonesia and provides a natural source of dietary fibre and antioxidants. This study evaluated the ability of green cincau, and other dietary fibres with or without the addition of anti-oxidant, epigallocatechin-3-gallate (EGCG), to prevent colorectal cancer in a 12 week azoxymethane (AOM) rat model. While all dietary treatments stimulated short chain fatty acid production (SCFA) in the digesta and faeces, no one treatment was able to significantly protect against aberrant crypt formation (ACF), when compared to the control diet. However, feeding green cincau leaves or extracts did not result in an increase in ACF compared to the control diet. Unexpectedly, when the dietary fibre source was pectin, 0.1% EGCG increased proliferative activity and liver lipid peroxidation when compared to the control diet containing cellulose. Examination of faecal microbial communities identified the presence of short chain acid producing bacteria, but a distinct community profile was not observed from any individual diet group. Overall, this research implies that combining dietary fibre with an antioxidant does not automatically equate to a beneficial response. Further work is required to investigate the health-promoting properties of green cincau.

## 1. Introduction

The incidence of colorectal cancer (CRC) is increasing in prosperous countries [1]. The westernised lifestyle is suggested as a major contributor to this disease [2], mainly due to diet, obesity, and inactivity [3]. The western diet tends to be high in red and processed meat and low in fruits and vegetables; all factors which may alter CRC risk. Fruit and vegetables are good sources of dietary fibre that may lead to a diminished risk of CRC by increasing stool bulk, decreasing transit time in the colon, diluting potential carcinogens, and inducing short chain fatty acid (SCFA) production [4,5]. In addition, fruit and vegetables contain a large number of potentially anti-carcinogenic agents including carotenoids, Vitamins C and E, selenium, and phenolics that have the ability to induce detoxification enzymes or form anti-neoplastic agents (compounds that prevent or inhibit the maturation and proliferation of neoplasms), and/or antioxidant effects [6,7]. Thus, fruit and vegetable consumption offers a unique combination of dietary fibre and antioxidants which could play a role in lowering CRC risk [8,9,10,11].

While some antioxidants are absorbed in the small intestine, unabsorbed antioxidants reach the colon for further metabolism by microbiota [12]. Previous research indicates that dietary fibre induces colonic metabolism of the phenolic compounds linked/trapped by dietary fibre [13,14]. However, there is limited evidence on the effects of combinations of specific dietary fibres and antioxidants on colon cancer. There is a possibility that combinations of different fibres and antioxidants may exert a synergistic or antagonistic effect.

The tropical plant *Premna oblongifolia* Merr. or green cincau from the *Verbenaceae* family is often used as the basis for Indonesian traditional food. Green leaves from this plant are dried, and then hot water extracts are prepared which contain 20% pectin as the main dietary fibre [15]. Research has shown that these extracts have free radical scavenging activity [15] and the ability to induce cell-mediated immune responses in vitro [16]. As a dietary fibre, the extract also has laxative properties, and it effectively induces the growth of lactic acid bacteria in the colon [17]. Using an in vitro fermentation model, we recently demonstrated the ability of green cincau extracts to stimulate total short chain fatty acid (SFCA) production and to kill colon cancer cells [18]. As the green cincau leaf extract contains both high pectin levels and phenolic compounds which give it antioxidant activities, this recent study aimed to compare the efficacy of this traditional source of dietary fibre and antioxidant with other dietary fibre, antioxidant mixtures in preventing colon cancer using an in vivo rat model.

The cell wall of fruits and vegetables generally consist of middle lamella, composed predominantly of pectic substances and the primary wall mostly contains cellulose [19]. When compared to cellulose in vitro [20] or inulin and lactitol in vivo [21], pectin is readily fermented by the colonic microbiota and produces higher levels of SCFA. However, the SCFA composition consists of a high proportion of acetic acid [22]. In vivo pectin has been shown to significantly suppress the formation of azoxymethane (AOM)-induced aberrant crypt foci (ACF), as well as crypt multiplicity and number of ACF cm^−2^ in rats fed a low fat (5%) diet [22]. Pectin also inhibited increased cell proliferation and crypt length in transmissible murine colonic hyperplasia induced by *Cytrobacter rodentium* [23].

Epigallocatechin-3-gallate (EGCG) is the major anti-oxidative polyphenolic compound in green tea [24]. Using the colitis-AOM model, when green tea and EGCG are included in the diet, they have been shown to inhibit the initiation, promotion, and progression stages of tumorigenesis [25]. In vitro EGCG has also been shown to hinder sphere formation in HepG2 and HCT-116 cell lines [26]. Combinations of EGCG with curcumin [27] or sulindac [28] or selenium [29] produced a synergistic effect inhibiting CRC growth. Most trials examining the effects of EGCG on CRC have been carried out in rats fed a cellulose or standard diet [29,30,31,32], therefore, it is interesting to assess whether substitution of the cellulose with pectin as the dietary fibre source will modulate the efficacy of EGCG.

Diets have indirect effects on gastrointestinal function of the host through the composition and activity of the human gut microbiota as well as the gut environment [3,33]. Substrate availability for gut bacteria is the major determinant of microbial community complexity and metabolites in the intestine [33]. Therefore, specific dietary interventions will change the type of bacterial groups dominating the colon [33,34]. The microbiota plays a significant role in metabolism of phenolic compounds in the colon [35,36] and phenolic bioavailability [37]. Phenolic compounds are degraded by colon microflora resulting in simpler derivative compounds, and the type of metabolites are dependent on the bacterial species present [35]. Fermentation by specific probiotic strains can also lead to a significant increase of free phenolic acids, therefore, improving phenolic acid bioavailability [36,38]. As the diversity of colon microbiota will be affected by dietary fibre, it is hypothesised that green cincau and pectin will have different effects on phenolic metabolism and bioactivities. The aim of this research was to evaluate and compare the effects of diets containing green cincau to those containing pectin or the control diet cellulose, with and without an EGCG antioxidant sources, on SCFA production, AOM-induced colonic ACF formation, and gut microbiota. A rat model of colorectal cancer is employed to test hypotheses.

## 2. Results

### 2.1. Body and Liver Weight Changes, Food and Water Intake

**Figure 1:**
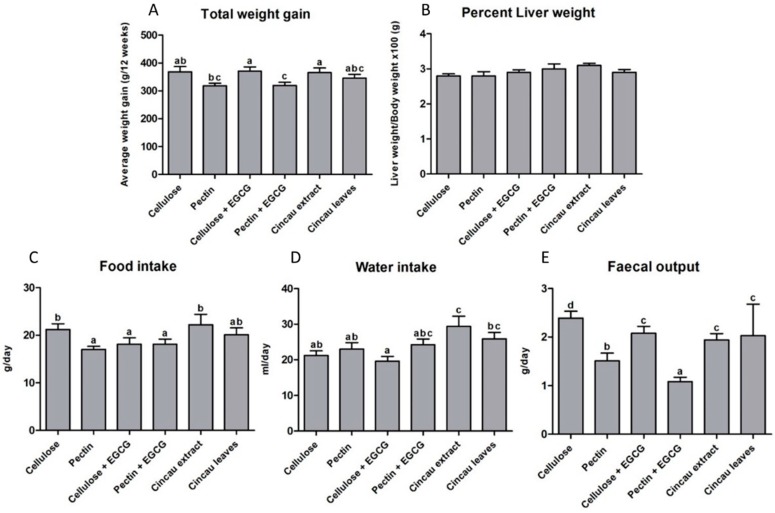
Effects of different dietary fibre diets on body weight gain (**A**), percent liver weight (**B**), food and water intake (**C**,**D**), and faecal output (**E**). Means with a different superscript (letters on the bar) are statistically significantly different when the LSD method was applied for post-hoc analysis *p* ≤ 0.05 (*n* = 12). EGCG, 0.1% Epigallocatechin-3-gallate.

Weight gains were similar between rats fed the control diet of cellulose only, or green cincau extract or cincau leaves as the dietary fibre. Pectin, as the dietary fibre source lowered body weight gain, but this difference only reached significance when 0.1% EGCG was added to the drinking water (*p* ≤ 0.05) (Figure 1). No differences in liver weight were observed between the groups. Data from rats housed in metabolic cages for 48 h indicated that the highest daily food intake was observed when rats were fed a diet containing green cincau extract or leaves or the cellulose control diet (Figure 1). In addition, green cincau extract or leaves also significantly increased water intake compared to both diets containing cellulose and pectin only. In contrast, the addition of antioxidant (EGCG) to the drinking water of animals on cellulose dietary fibre significantly reduced food intake. The addition of 0.1% EGCG to either a cellulose or pectin based fibre diet had no effect on water intake. Pectin had a reducing effect on faecal output, and when 0.1% EGCG was added to the drinking water, faecal output further declined (Figure 1).

### 2.2. Short Chain Fatty Acids (SCFAs)

The diets containing different dietary fibre with and without antioxidant led to differences in SCFA concentrations (acetate, butyrate, and proponiate) being detected in colon digesta and faeces collected from rats (Table 1). SCFA concentrations levels were almost double in the digesta compared to faeces. The pectin diet significantly increased acetate, propionate, butyrate and total SCFA concentrations in both the faeces and the digesta compared to the control cellulose diet. Diets containing green cincau extract and leaves also increased acetate, butyrate, and total SCFA concentrations but not propionate in the faeces when compared to the control cellulose diet. In the digesta, the green cincau leaf diet (but not extract) led to a significant increase in acetate levels compared to the cellulose group, but no other SCFA differences were observed. The addition of 0.1% EGCG to the drinking water of rats on the pectin diet significantly lowered acetate, propionate, and total SCFA but not butyrate levels in the digesta, and had no significant effects on SCFA levels in the faeces when compared to the pectin only diet. In contrast, when the dietary fibre source was cellulose, the addition of 0.1% EGCG drinking water increased acetate and butyrate levels in the digesta, but also had no significant effect on faecal SCFA levels. In most cases, the pH was significantly lower in the digesta and faeces of rats fed all diets when compared to the cellulose control diet.

### 2.3. Aberrant Crypt Foci (ACF) Formation

Aberrant crypt foci (ACF) were observed in the colon of all rats fed either the control cellulose diet or diets containing other dietary fibre six weeks after the second AOM injection was used to induce these early histopathological detectable signs which can lead to colorectal cancer (Table 2). ACF were present predominantly in the distal and middle colon but were also observed at low levels in the proximal colon. The total number of ACF in the colon, the total number of crypt foci <4 and the number of single crypt foci was higher in rats fed the pectin as the main dietary fibre (*p* ≤ 0.05) compared to control rats fed cellulose (Table 2). There was no significant difference in total ACF between rats fed either cincau extract or cincau leaves in comparison to the control cellulose diet. The addition of 0.1% EGCG drinking water to the diet had no significant effects on total ACFs or number of foci when either cellulose or pectin was the dietary fibre.

### 2.4. Cell Proliferation in Distal Colon

**Figure 2:**
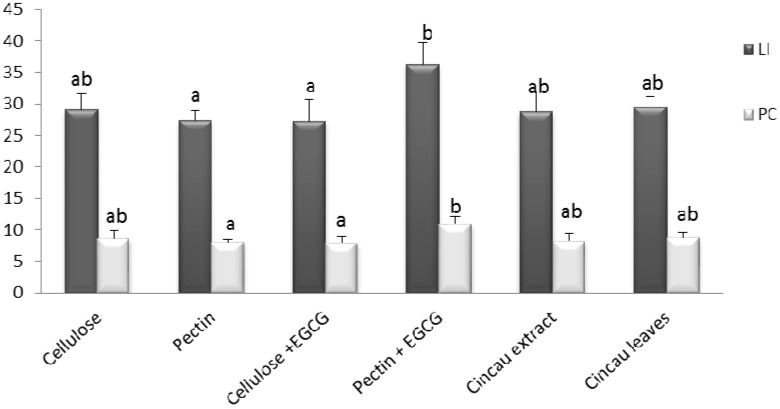
Effects of different fibre diets on proliferating cell nuclear antigen (PCNA) labelling index (dark bars) and the number of PCNA positive cells (light bars) in the mucosa of distal colon of rats six weeks after the second injection with the carcinogen azoxymethane (AOM) (2 × 15 mg/kg). Values are the mean ± SE (*n* = 12). Means with a different letters on the bar are statistically significantly different (*p* ≤ 0.05). EGCG, 0.1% epigallocatechin-3-gallate. LI, the PCNA labelling index (%); PC, the number of PCNA positive cells.

Cell proliferation was evaluated by assessing proliferating cell nuclear antigen (PCNA) staining in the distal colonic crypts. There were no differences in PCNA labelling index or positive cells between the control diet cellulose and the pectin and cincau dietary fibre groups. However, when the pectin fed rats were supplemented with 0.1% EGCG drinking water, there was a significant increase in PCNA labelling index and the number of PCNA positive cells (*p* ≤ 0.05) (Figure 2). In contrast, the EGCG drinking water had no effect on proliferation when added to the cellulose dietary fibre group.

### 2.5. Lipid Peroxidation in Liver

**Figure 3:**
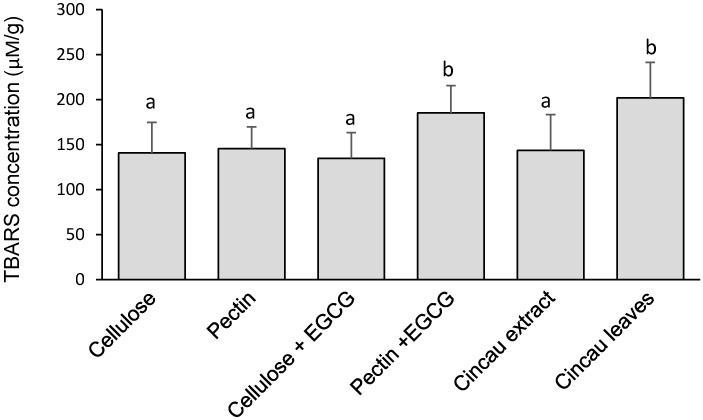
Effects of different diets on thiobarbituric acid reactive substance (TBARS) concentration in the livers of rats six weeks after the second AOM injection (2 × 15 mg/kg). Values are the mean ± SE (*n* = 10). Means with a different letters on the bar are statistically significantly different (*p* ≤ 0.05). EGCG, 0.1% epigallocatechin-3-gallate.

Thiobarbituric acid reactive substance (TBARS) concentrations may serve as a biochemical marker of oxidative stress in CRC [39,40,41]. Rats fed green cincau leaves had significantly higher liver TBARS levels compared to the control diet cellulose, or the pectin and cincau extract diets. In addition, when 0.1% EGCG was given in drinking water to the pectin dietary fibre group, liver TBARS was significantly increased (*p* ≤ 0.05) (Figure 3). In contrast, the EGCG drinking water had no effect on TBARS level in the rats fed cellulose as the dietary fibre.

### 2.6. Microbial Profile of the Colon Digesta

**Figure 4:**
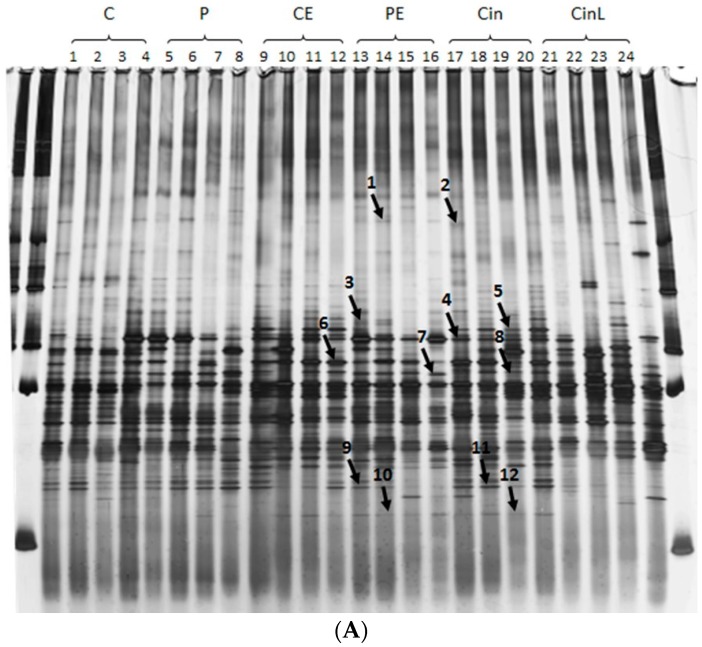

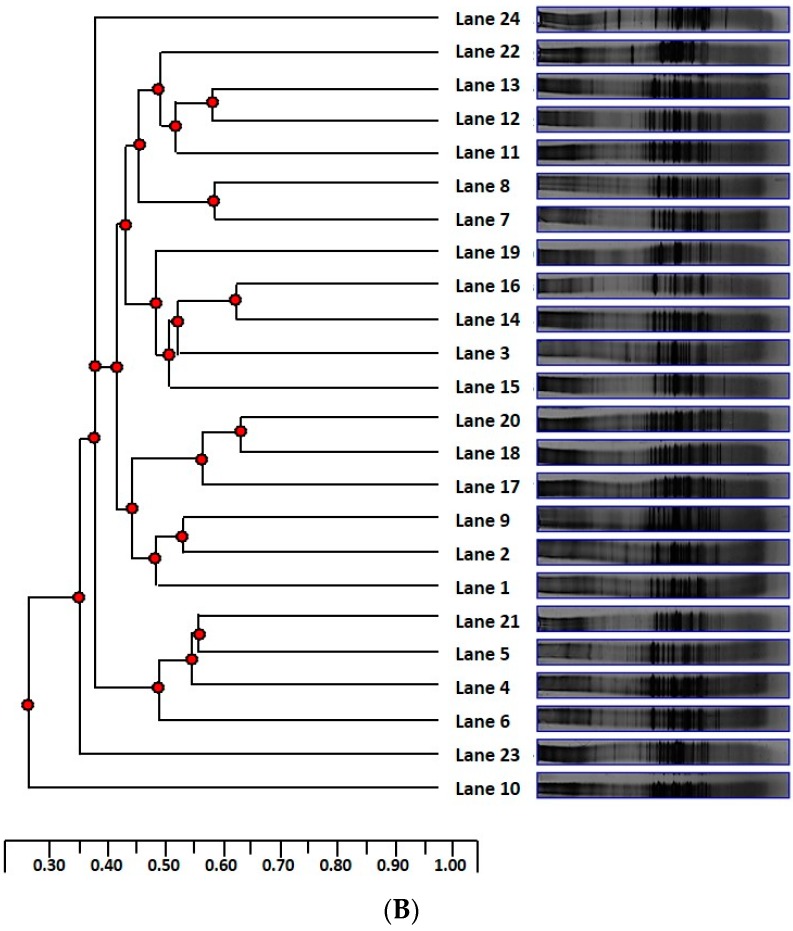
Denaturing gradient gel electrophoresis (DGGE) analysis (**A**) and unweighted pair groups with mathematical averages (UPGMA) dendrogram (**B**) of 16S rRNA gene fragments representing the bacterial population amplified from DNA extracted from digesta from rats six weeks after the second AOM injection (2 × 15 mg/kg). (**A**) Bands of interest are indicated by arrows and numbered 1 to 12. Bands 1 to 7 were cut out for DNA extraction and sequencing. (**B**) Scale refers to similarity index Lane 1–4: C = cellulose; Lane 5–8: P = pectin; Lane 9–12: CE = cellulose + EGCG; Lane 13–16: PE = pectin + EGCG; Lane 17–20: Cin = cincau extract; Lane 21–24: CinL = Cincau leaves. EGCG, 0.1% epigallocatechin-3-gallate.

The microbial communities in the digesta of rats were examined by utilising 18s RNA PCR and denaturing gradient gel electrophoresis (DGGE) (Figure 4a). Considering the similarity index of those lanes with values higher than 0.4, four distinct clusters (cluster one including lane 7, 8, 11, 12, 13, and 22; cluster two including lane 15, 3, 14, 16, and 19; cluster three including lane 1, 2, 9, 17, 18, and 20; cluster four including lane 6, 4, 5, and 21) (Figure 4b) were seen. However, no distinct cluster based on dietary fibre or antioxidant source was observed. Sequencing of selected bands confirmed the presence of species such as *Clostridiales* sp. SM4/1 and *Clostridium saccharolyticum* that commonly grow in the colon and produce SCFA and *Oscillibacter valericigenes* a valeric acid producer [42] (Figure 4 and Table 3).

Band 2 and 7 were visualised at different mobility and size on the DDGE gel, but both identified a closest match at 99 and 100% sequence identity respectively with the lactic acid producing bacteria *Lactobacillus amylolyticus* [44]. Likewise, bands 4 and 6 were identified as different sized bands on the DDGE, but both matched to the butryate producer *Clostridiales* sp. SM4/1 [46].

## 3. Discussion

Green cincau is a traditional food source in Indonesia, providing a natural source of dietary fibre and antioxidants. To our knowledge, this is the first study to assess the effects of this popular food in vivo. ACF formation, an early neoplastic marker and SCFA production using the AOM CRC rat model were employed to study the efficacy of green cincau, and both cincau extract and cincau leaves had similar effects to those of the control diet with cellulose as the main source of dietary fibre. More importantly, no detrimental effects of this indigenous food source were observed. In contrast, when pectin was utilised as the main dietary fibre, ACF number and their size increased significantly. In addition, when 0.1% of the antioxidant, EGCG was added to drinking water, either in combination with cellulose or pectin, it was unable to significantly protect against ACF formation. In fact, 0.1% EGCG increased proliferation in the distal colon of rats fed pectin as their dietary fibre source. While introducing pectin as the main dietary fibre led to a significant increase in acetate, propionate, and butyrate levels in both the digesta and in faeces, in this model the SCFA increase did not provide any protection against ACF formation.

The cincau extract contained approximately 46% insoluble non-starch polysaccharides (NSPs) and 6% soluble NSPs [18], and this may explain why its effects on ACF formation mimicked those observed with rats fed insoluble cellulose as the main dietary fibre. Although gram for gram (when green cincau was used as the dietary fibre), only half the amount of insoluble cellulose would have been ingested by rats compared to the cellulose groups. Despite the lower levels of insoluble fibre ingested by the green cincau groups, levels of acetate, butyrate, and total SCFA levels were significantly higher in faeces, suggesting some other component other than insoluble fibre in green cincau was able to stimulate SCFA production. It has previously been demonstrated in animal models that increasing ingestion of butyrylated or resistant starches leads to increased butyrate levels in the colon which in turn protects against AOM induced neoplasms [47,48]. It has also been observed that a diet containing ten percent inulin or pectin stimulates SCFA production in the cecum of rats [20]. It is possible that cincau contains some resistant starches, inulin, or other components that may also contribute to SCFA production. However, long chain polysaccharide analysis of these cincau extracts determined that they only contain 2.5 ± 0.4% long chain polysaccharides compared to the 92.3 ± 2.7% detected in inulin purified from chicory (Stangoulis J.C.R, Flinders University, Adelaide, South Australia, Personal communication, 2016). Thus, while it is still unclear what component of green cincau is adding to SCFA production, it appears to be present in both the extract and the leaves. The increase in SCFA produced by green cincau fermentation compared to cellulose was only observed in the faeces and not higher up in the colon, and this may be why the increased SCFA levels did not offer protection against early neoplasms in the colon.

Both green tea and grape seed extract contain polyphenols and are protective against CRC when ingested in the rat AOM model [29,49]. Likewise, green cincau leaves are reported to contain polyphenols with antioxidant properties [50,51], yet it is unknown whether the cincau extracts utilised in this study contained or retained bioactive polyphenols. In this study, extracts were dried in an oven and this may have resulted in a partial loss of the phytochemical bioactive compound, as reported elsewhere [52]. However, in this study, the effect of green cincau was still evident with this treatment procedure and regular ingestion of cincau leaf extract could protect against CRC if the phytochemical compounds in cincau are not lost during traditional processing [53]. Green cincau leaves also contain chlorophyll and increasing chlorophyll intake has previously been shown to reduce the risk of CRC in men [54]. Previous studies have shown that chlorophyll is preserved better using freeze-drying methods over oven-based ones [55]. Further evidence that oven drying may lead to the deterioration of the extracts in this study is provided by the observation that liver TBARS level in rats ingesting the oven dried cincau leaves were significantly higher when compared to those in rats feeding on cincau extract or the control cellulose diet. Any toxic by-products created during drying may have induced stress in the liver and also led to a masking of any protective effects that may be possible with this food source. This finding highlights the importance of optimizing the extraction process to produce the maximum yield of bioactive compounds when studying natural products such as green cincau. Despite the lack of anti-cancer protective effects observed, it is important to note that no detrimental effects of green cincau on ACF formation or cell proliferation were observed. This finding in itself suggests that further investigation of green cincau as a functional food is warranted.

Green leaf extracts from the cincau plant contain about 20% pectin [15]. In this study, cincau extracts contained approximately 6% insoluble fibre, so considerably less pectin than traditionally observed and this may be why cincau effects were quite different to pectin. Whilst the pectin only diet enhanced the production of total SCFA including acetate, propionate, and butyrate in the digesta and faeces, unexpectedly it led to an increase in cell proliferation and increased the number of neoplastic lesions measured as ACFs when compared to the cellulose only control diet. The stimulatory effect of pectin on SCFA production observed in the digesta is consistent with the literature. Pectin is a soluble dietary fibre and is more fermentable than cellulose [56]. Pectin, but not cellulose was shown to increase butyrate levels in faecal water when fed to BalBc mice [57] and increased caecal SCFA when fed to rats [20]. However, these stimulatory effects on SCFA production are normally accompanied by a decrease in ACF number [20] and a reduction in tumor size and volume [58,59]. There is an abundance of literature suggesting that increased SCFA production protects against preneoplastic lesions [60]. The increase in butyrate levels in the colon is thought to lead to the upregulation of the proapoptotic caspase 3 and downregulation of proapoptotic bcl-2 family to reduce tumor size [58]. In this study, despite pectin stimulating SCFA production, it led to an increase in proliferation within the colon and an increase in preneoplastic lesions, thus, appeared to be acting more like a pro-carcinogen. The diet fed to rats only contained 5% pectin, so it is possible that more pectin needs to be consumed by rats to act as a protective, or that pectin needs to be delivered with other nutrients or fibre sources to be protective.

EGCG is the major anti-oxidative polyphenolic compound in green tea [24] and has been found to inhibit tumorigenesis during the initiation, promotion, and progression stages [25]. Most trials examining the effects of EGCG on CRC have been carried out in rats fed diets containing cellulose or a standard diet [29,30,31,32]. Previously, 1% and 2% (*w*/*v*) tea infusion reduced the number of ACF as well as liver and colon lipid peroxidation significantly [31]. Not all phenolic compounds suppress SCFA production in the colon; some are capable of upregulating SCFA production [61], and some of them have no effect. Our results suggest the effect of EGCG or its metabolites depends on the fermentability of the dietary fibre included in the diet. The addition of 0.1% EGCG in the drinking water increased SCFA production when the fibre was cellulose but decreased SCFA production when the fibre was pectin. When the rats fed pectin were also given 0.1% EGCG in the drinking water, it increased liver TBARS levels indicating this dose was leading to oxidative stress in the liver. This did not occur when the dietary fibre was cellulose.

It is also worth noting that a recent report by an expert panel from the European Food Safety Authority concluded that there is evidence that intake of EGCG doses equal or above 800 mg/day in the diet may lead to a degree of hepatoxicity as measured by increases in serum transaminase level in a small proportion of the population [62]. Thus, caution must be taken when considering adding high levels of EGCG to the diet for health benefits. The rats in this study were drinking approximately 20 mL of water per day, containing 0.1 g EGCG per 100 mL water which equates to a relatively high dose of 20 mg EGCG/day for a 500 g rat. However, it did not appear to be protecting against ACF formation when combined with either fibre.

The cancer preventative effects of the antioxidant EGCG that have been previously reported are thought to work through direct anti-oxidant or indigenous anti-oxidant induction activities [63]. However, there have been indications that EGCG causes oxidative damage to isolated and cellular DNA due to induction of H_2_O_2_ production [64]. EGCG also induced pro-MMP-7 expression via O_2_-production in HT-29 and Caco-2 cell lines [65]. The addition of pectin increases the concentration of bile acids (deoxycholic acid and lithocholic acid) in comparison to a standard diet [66]. These acids, especially deoxycholic acid, are able to impair mitochondrial function by causing lipid peroxidation, and also induce free radical production in isolated rat hepatocytes [67]. In addition, they have been shown to induce lipid peroxidation in rat liver [68]. In our animal study, pectin administration may have increased the concentration of pro-oxidant bile acid in the colon, resulting in the production of free radicals that are able to oxidise EGCG. As a result, EGCG resulted in a pro-oxidant activity and this decreased total SCFA in the digesta and increased cell proliferation in the distal colon, but this did not lead to differences in ACF number when compared to the pectin only diet [69].

Microbial profiling was utilised to examine whether different dietary fibre led to different microbial communities. While several community clusters were identified, these did not cluster according to dietary fibre or the addition of anti-oxidant. Composition of human colon bacteria is individual [34], therefore, when subjects ingest a specific food, a specific metabolite profile differing in composition and concentration will be displayed by each subject [70]. In our study, dietary fibre alone and dietary fibre mixed with EGCG intake did not exhibit distinct colon microbial profile patterns (Figure 4) suggesting an inter-individual variation of the initial composition of the gut microbiota in each rat as is observed for humans [34]. Some bands appear to be dominant in response to dietary fibre or antioxidant. This indicates that, even though the rat colon microbial communities display individual variation, dietary fibre and EGCG intake produces marked changes in the gut microbiota [34,71]. Despite the variation observed, several bands were identified in rats fed different diets. DNA from both butyrate producing bacteria (*Clostridiales* sp. SM4/1) and acetic acid producing bacteria (*Lactobacillus amylolyticus*) were identified in the digesta of rats fed pectin, suggesting that this fibre may have promoted the growth of microbes that preferentially produce SCFA. The dominant microbial community in the colon depends on the type of the available substrate and the gut environment [72]. Cellulose and pectin have different fermentability [56] and induce a different microbial profile when they are administered to the rats. As a consequence, when EGCG is given to rats fed cellulose or pectin, it may be metabolised by a different microbial community in the rat colon depending on the diet. There are some bacterial groups that produce acetate, propionate, and butyrate, [71] but this will change in response to specific dietary interventions [33,34]. Therefore, this study suggests that the microbes producing SCFA in rats fed cellulose and rats fed pectin are different species and, therefore, they respond differently to EGCG or its metabolites. Whilst the green cincau extracts contained polyphenols which are known to affect gut microbiota composition [73], no profile or bands specific to the two green cincau rat groups was observed.

## 4. Materials and Methods

### 4.1. Materials

All chemicals were purchased from Sigma-Aldrich Chemical Co. (St Louis, MO, USA) unless otherwise stated.

### 4.2. Green Cincau Leaf Preparation

Green cincau leaves (*Premna oblongifolia* Merr.) were collected from traditional farmers in Indonesia as described in Reference [18]. Fresh cincau leaves were dried at 50 °C (water content around 12%) and ground into fine powder before being imported into Australia using AQIS permit (IP07024278). Cincau extracts were prepared by adding hot water to dry cincau leaf powder (5 g per 100 mL) and then stirred for 5 min at maximum speed. The mixture was then filtered, allowed to set at room temperature, and the resulting jelly-like extract was oven dried at 50 °C [15]. Compositional analysis of similar hot water extracts determined they contained 5.8% soluble non-starch polysaccharides (NSPs) and 46.3% insoluble NSPs [18]

### 4.3. Animals and Diet

All animal work was approved by the Flinders University Animal Welfare committee under ethics application 761/2010 (approved 23 September 2010), and male Sprague Dawley rats were obtained from the Animal Resource Centre, Perth, Western Australia. The animals, diets and the experimental procedure were prepared as described by Le Leu et al. [74]. Dietary fibre was added into the experimental diet based on the AIN-76A standard for purified diets for rats and mice (Report of the American Institute of Nutrition ad hoc Committee on Standards for Nutritional Studies, 1977). EGCG (Sigma-Aldrich Chemical Co., St Louis, MO, USA) was given in tap water at 0.1% EGCG concentration as this dose had previously been shown to reduce total ACF number [75]. Each group of rats (*n* = 12) was fed one of six diets (Table 4) including a control diet containing the standard cellulose only diet. Animals were given water and food ad libitum.

After 4 weeks on experimental diets, rats were housed in metabolic cages for 48 h for measuring food intake and faecal output. At the beginning of week 5, each rat received a subcutaneous injection of AOM (15 mg/kg body weight) once weekly for two weeks. Rats were maintained on their experimental diets for six weeks after the second AOM injection. Fresh faecal samples were collected for SCFA analysis one week before the trial ended. At the termination of the study (12 weeks), rats were anaesthetised and killed. After this, laparotomy was performed, and the large intestine was removed. The intestines were opened longitudinally, and the contents were emptied, and the colon was fixed flat as described below. Caecal digesta was collected for analysis of SCFA concentrations. Liver tissue was also collected for thiobarbituric acid reactive substance (TBARS) measurements.

### 4.4. Determination of SCFA Composition of Faecal and Caecal Digesta Samples

Faecal and caecal digesta samples were collected and immediately diluted in three volumes of internal standard solution (heptanoic acid, 1.68 mM/L) before storage at −20 °C to protect SCFAs from degradation. For SCFA measurements samples were thawed and centrifuged at 3000× *g* for 10 min. Each supernatant was distilled and subjected to gas chromatography (HewlettPackard 5890 Series II A with a Zebron column, ZB-FFAP, 30 m × 0.53 mm i.d, 1-µm film) as previously described [76]. The results are expressed as µM/g of sample and a standard SCFA mixture containing acetate, propionate, and butyrate was used for these calculations.

### 4.5. Measuring ACF Number and Multiplicity

Colons were fixed flat in 10% buffered formalin solution containing 3.6% formaldehyde for 24 h then transferred to 70% ethanol for histological processing. The mucosal surface of the colons was stained with 0.2% methylene blue, and the number of ACF was counted under a light microscope [77]. Colons were divided into proximal, medium distal, and distal sections. The proximal site was indicated by the herringbone region, and the distal was divided into medium distal and distal by halving the colon without proximal region.

### 4.6. Proliferating Cell Nuclear Antigen (PCNA) Staining

Standard immunohistochemical techniques were used to detect proliferating cell nuclear antigen (PCNA) as a marker of both the number and the distribution of proliferating cells in colonic crypts [48]. Briefly, deparaffinised sections were rehydrated in a graded series of ethanol from 100% to 50% and then to distilled water. Antigen retrieval using a pressure cooker in 0.01 M sodium citrate buffer was applied for 1 h. The primary PCNA mouse monoclonal antibody (PC 10, Santa Cruz Biotechnology, Santa Cruz, CA, USA) was placed on the slides (1/500 dilution) and incubated overnight at room temperature. A Level 2 Ultra Streptavidin detection system (Signet Laboratories, Inc., Dedham, MA, USA) utilizing a biotinylated goat anti-mouse as the secondary antibody was used for detecting PCNA positive cells. The slides were counterstained for one min with haematoxylin. The cell number along the crypt and the number of PCNA positive cells were counted randomly under light microscopy (magnification 40×). The PCNA labelling index (LI) was calculated as the number of positive cells divided by the total number of cells in each crypt column.

### 4.7. Measuring Lipid Peroxidation in Rat Liver

Lipid peroxidation in rat liver was quantified by measuring malondialdehyde levels in the liver via the thiobarbituric acid reactive substances (TBARS) method [78]. Malondialdehyde in the liver reacts with thiobarbituric acid to produce a pink colored complex with a peak absorbance at 532 nm. A TBARS standard curve was produced using 1,1,3,3-tetraethoxypropane as the TBARS source, and then lipid peroxidation levels were expressed as µM TBARS per gram liver [78].

### 4.8. Measuring Faecal Bacterial Community

#### 4.8.1. Extraction of Bacterial DNA from Rat Faecal Samples

For DNA extraction, faecal samples were diluted 1:4 (*w*/*v*) in 0.98% sodium chloride solution. DNA was extracted from about 500 mg diluted samples using PowerSoil^®^ DNA Isolation Kit, a soil DNA extraction kit (MO BIO Laboratories, Carlsbad, CA, USA) as per the manufacturer’s protocol.

#### 4.8.2. Bacterial 16S rDNA Amplification

16Sr genes were amplified by PCR with universal bacterial primers 341FGC (5′-CGCCCGCCGCGCGCGGCGGGCGGGGCGGGGGCACGGGGGGCCTACGGGAGGCAGCAG-3′) and 518R (5′-ATTACCGCGGCTGCTGG-3′) [79]. A mastermix containing 2 µL of purified DNA (145–371 ng/µL), 0.2 mM dNTPs, 1× GoTaq^®^ Flexi buffer (Promega, Madison, WI, USA), 1.5 mM MgCl_2_, 0.025 Units GoTaq^®^ Flexi Polymerase, 20 pM each of the forward and reverse primers, 341FGC and 518R respectively, and sterile water was prepared for PCR amplification. The thermocycling program consisted of 1 cycle of 5 min at 95 °C; 30 cycles of 1 min at 95 °C, 1 min at 65 °C, 1.5 min at 72 °C; and a final extension at 72 °C for 10 min [80].

#### 4.8.3. Denaturing Gradient Gel Electrophoresis (DGGE)

Bacteria in the colonic digesta were assessed using DGGE following the method described by Pérez-Leblic et al. [81]. DGGE was performed with a D-code Universal Mutation Detection System (Bio Rad, Hercules, CA, USA). PCR products were loaded onto 6% polyacrylamide gels (Bio Rad) containing a formamide-urea linear denaturing gradient of 25–65%. Gels were run in 1× TAE at a constant voltage of 60 V for 18 h at 60 °C. Bands were visualised by staining the gels with silver nitrate solution (12.5%). The gels were exposed to UV light to visualise the bands and digitalised in a Gel Doc 2000 (Bio Rad). Digitised gel images were then analysed with TotalLab analysis package (TotalLab Ltd., NE1 2JE, Newcastle upon Tyne, UK). TotalLab software was used to produce unweighted pair groups with mathematical averages (UPGMA) dendrograms and the Shannon–Weaver Diversity Index (Ho) was used to establish bacterial community diversity.

#### 4.8.4. Excision, Cloning, and Sequencing of Selected Bands from DGGE Gels

Selected bands were excised from DGGE gels with a sterile razor, placed in 40 μL sterile water and incubated at 4 °C for diffusion of DNA into the water. The bands were chosen based on their appearance intensities in DGGE. The DNA was cloned into pGEM^®^-T Easy Vector Cloning Kit (Promega) according to the manufacturer’s protocol. Competent JM109 E. coli cells were transformed and plated. Plasmid DNA was purified with Wizard^®^ Plus SV Minipreps (Promega) and quantified with Nanodrop (Thermoscientific, Waltham, MA, USA) from colonies containing inserts. The DNA samples were then sent for sequencing to AGRF (Australian Genome Research Facility) according to AGRF requirements (www.agrf.org.au). The sequences obtained were compared to available database sequences for bacteria using the Basic Local Alignment Search Tool (BLAST) (http://blast.ncbi.nlm.nih.gov/). Sequences with identity >95% were considered to represent the same taxonomic group.

### 4.9. Statistical Methods

Results are expressed as the mean ± SE. Statistical analysis was carried out with the statistical program SPSS version 19. One way-analysis of variance (ANOVA) was used to analyze the variation between groups. The Least Significant Difference (LSD) test was used for post-hoc analysis. Results were considered significant if *p* ≤ 0.05.

## 5. Conclusions

In conclusion, our in vivo results show that consuming fibre and antioxidant together in the diet does not always lead to positive synergistic effects in inhibiting CRC development. Combination of pectin and EGCG, a polyphenolic compound extracted from green tea inhibited the ability of pectin to stimulate SCFA production in digesta and increased cell proliferation in the distal colon. These detrimental effects were accompanied with increasing lipid peroxidation in the liver. In contrast, this study demonstrates for the first time that traditional dried green cincau extract which contains pectin and antioxidants does not increase total ACF number compared to control cellulose diet, i.e., did not increase CRC risk. Overall, this research implies that the beneficial effect of combining a dietary fibre with antioxidant does not automatically equate to their individual effect, but their combined effect depends on interactions between the fibre and the existing colon microbial community. Natural dietary fibre and antioxidant sources (as found in fruits, vegetables, and plant extracts) may exhibit a protective effect against CRC, and when utilizing purified sources of these compounds to create functional food care should be taken during extraction or processing to protect their potency. Ideally, consumption of fresh dietary fibre and antioxidants sources is most likely to give the greatest protection against CRC.

## Figures and Tables

**Table 1:** Effects of different diets on short chain fatty acids (SCFA) concentration (µM/g) and pH in the digesta and faeces from rats six weeks after the second injection with the carcinogen azoxymethane (AOM) (2 × 15 mg/kg) (*n* = 12).

Type	Cellulose Only (Control Diet)	Pectin	Cellulose + EGCG	Pectin + EGCG	Cincau Extract	Cincau Leaves
Digesta						
Total SCFA	45.9 ± 8.4 ^ab^	107.3 ± 12.2 ^c^	69.1 ± 5.3 ^b^	65.8 ± 7.1 ^b^	44.9 ± 5.2 ^a^	64.5 ± 10.5 ^ab^
Acetate	18.2 ± 3.2 ^a^	40.0 ± 3.8 ^c^	28.2 ± 2.1 ^b^	26.2 ± 2.8 ^ab^	19.1 ± 2.0 ^ab^	28.3 ± 5.3 ^b^
Propionate	5.0 ± 1.0 ^ab^	16.0 ± 1.9 ^d^	7.8 ± 0.8 ^bc^	9.2 ± 1.0 ^c^	4.0 ± 0.5 ^a^	5.7 ± 1.1 ^ab^
Butyrate	4.2 ± 0.9 ^a^	9.8 ± 1.4 ^c^	7.5 ± 0.7 ^bc^	6.9 ± 1.7 ^abc^	5.0 ± 0.8 ^ab^	6.5 ± 1.0 ^abc^
pH	6.9 ± 0.06 ^a^	6.7 ± 0.08 ^b^	6.7 ± 0.05 ^b^	6.7 ± 0.07 ^b^	6.7 ± 0.05 ^b^	6.7 ± 0.05 ^b^
Faeces						
Total SCFA	14.8 ± 2.0 ^a^	44.4 ± 8.3 ^cd^	17.5 ± 2.4 ^ab^	51.9 ± 9.0 ^d^	30.8 ± 3.9 ^bc^	33.7 ± 4.2 ^c^
Acetate	5.8 ± 0.6 ^a^	14.5 ± 3.0 ^c^	7.2 ± 0.9 ^ab^	14.5 ± 2.2 ^c^	12.6 ± 1.1 ^bc^	13.7 ± 1.2 ^c^
Propionate	1.4 ± 0.2 ^a^	8.0 ± 1.3 ^b^	1.9 ± 0.3 ^a^	7.3 ± 1.0 ^b^	2.3 ± 0.2 ^a^	2.8 ± 0.3^a^
Butyrate	1.6 ± 0.2 ^a^	4.7 ± 0.5 ^b^	2.4 ± 0.3 ^a^	6.3 ± 1.0 ^b^	4.6 ± 0.5 ^b^	4.8 ± 0.5 ^b^
pH	6.7 ± 0.06^a^	6.4 ± 0.05^c^	6.4 ± 0.04^c^	6.5 ± 0.05^bc^	6.6 ± 0.05 ^b^	6.4 ± 0.05^c^

Values are expressed as mean ± SE. Means in the same row with a different superscript are significantly different when the LSD method was applied for post-hoc analysis (p ≤ 0.05). EGCG, 0.1% epigallocatechin-3-gallate.

**Table 2:** Effects of different diets on formation of aberrant crypt foci (ACF) in rat colon six weeks after AOM injections (2 × 15 mg/kg) (*n* = 12).

Type/Location	Cellulose Only (Control Diet)	Pectin	Cellulose + EGCG	Pectin + EGCG	Cincau Extract	Cincau Leaves
ACF Incidence	12/12	12/12	12/12	12/12	12/12	12/12
Total No. ACF	73.2 ± 12.1 ^a^	110.7 ± 19.6 ^b^	64.7 ± 8.5 ^a^	114.5 ± 14.1 ^b^	79.7 ± 10.7 ^ab^	88.8 ± 10.7 ^ab^
1 Crypt	32.9 ± 5.2 ^a^	53.6 ± 10.9 ^b^	31.5 ± 4.5 ^a^	52.3 ± 5.6 ^b^	43.4 ± 4.3 ^ab^	44.5 ± 5.0 ^ab^
2 Crypts	26.8 ± 4.7 ^ac^	40.5 ± 6.7 ^ab^	23.7 ± 3.2 ^c^	41.3 ± 5.9 ^b^	23.5 ± 4.6 ^c^	30.2 ± 4.5 ^abc^
3 Crypts	8.4 ± 1.7 ^a^	10.6 ± 1.9 ^ab^	7.1 ± 1.3 ^a^	14.5 ± 2.5 ^b^	7.7 ± 2.1 ^a^	9.7 ± 1.8 ^ab^
<4 Crypts	68.2 ± 11.2 ^a^	104.9 ± 18.5 ^b^	62.3 ± 8.8 ^a^	108.2 ± 13.0 ^b^	74.7 ± 9.3 ^ab^	84.4 ± 9.8 ^ab^
≥4 Crypts	4.5 ± 1.0 ^ab^	5.8 ± 1.3 ^b^	2.4 ± 0.5 ^a^	6.4 ± 1.5 ^b^	4.3 ± 1.3 ^ab^	4.3 ± 0.9 ^ab^
Proximal Colon	1.6 ± 0.6 ^a^	2.0 ± 0.8 ^a^	0.6 ± 0.2 ^a^	3.6 ± 2.7 ^a^	3.2 ± 1.9 ^a^	2.4 ± 1.4 ^a^
Middle Colon	38.8 ± 6.4 ^a^	64.3 ± 14.3 ^a^	43.0 ± 7.2 ^a^	63.0 ± 11.3 ^a^	44.1 ± 9.7 ^a^	49.8 ± 8.1 ^a^
Distal Colon	32.8 ± 6.7 ^ac^	44.4 ± 6.5 ^ab^	21.2 ± 2.9 ^c^	48.0 ± 3.2 ^b^	32.4 ± 4.4 ^ac^	36.4 ± 6.8 ^ab^

Values are expressed as mean ± SE. Means in the same row with a different superscript are significantly different when the LSD method was applied for post-hoc analysis (p ≤ 0.05). EGCG, 0.1% epigallocatechin-3-gallate.

**Table 3:** Closest relatives of DNA sequences of bands excised after denaturing gradient gel electrophoresis (DGGE) in Figure 4. DNA was extracted, amplified and sequenced from the digesta of rats fed different diets, six weeks after the second AOM injection (2 × 15 mg/kg).

Band	Fragment Size	Closest Relative	Accession Number	Percent Similarity	Known Function
1	196	*Lactobacillus johnsonii* DPC 6026	NC017477.1	100	Probiotic [43]
2	196	*Lactobacillus amylolyticus*	ADNY01000006.1	99	Lactic acid producer [44]
3	173	*Oscillibacter valericigenes*	NC_016048	97	Valerat producer [42]
4	170	*Clostridiales* sp. SM4/1	FP929060.1	96	Butyrate producer (genomesonline.org)
5	173	*Clostridium saccharolyticum*	NC_014376.1	97	Acetic acid [45]
6	171	*Clostridiales* sp. SM4/1	FP929060.1	98	Butyrate producer (genomesonline.org)
7	177	*Lactobacillus amylolyticus*	ADNY01000006.1	100	Lactic acid producer [44]

**Table 4:** Composition of control and experimental diets.

(g/1000 g in Diet)	Cellulose Only (Control Diet)	Pectin	Cellulose + EGCG	Pectin + EGCG	Cincau Extract	Cincau Leaves
Casein	190	190	190	190	190	190
Corn Starch	430	430	430	430	430	430
Cellulose	50	-	50	-	-	
Pectin	-	50	-	50	-	-
Cincau extract	-	-	-	-	50	-
Cincau leave powder	-	-	-	-	-	50
Corn oil	180	180	180	180	180	180
Sucrose	109	109	109	109	109	109
dl-Methionine	3	3	3	3	3	3
Choline	1	1	1	1	1	1
Mineral Mix *	35	35	35	35	35	35
Vitamin Mix *	10	10	10	10	10	10
EGCG in water **	-	-	0.1%	0.1%	-	-

* AIN-76 vitamin and mineral mixtures with modified calcium at 0.5 mg/g, phosphorus at 3.6 mg/g, folic acid at 0.23 mg/g, and vitamin D3 at 0.11 IU/g, ** EGCG, 0.1% epigallocatechin-3-gallate.

## Abbreviations

ACF	Aberrant crypt foci
AOM	Azoxymethane
CRC	Colorectal cancer
DDGE	Denaturing gradient gel electrophoresis
EGCG	Epigallocatechin-3-gallate
TBARS	Thiobarbituric acid reactive substances
SCFAs	Short chain fatty acids
